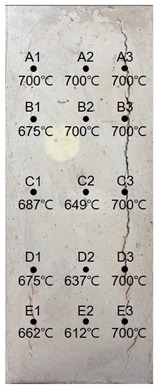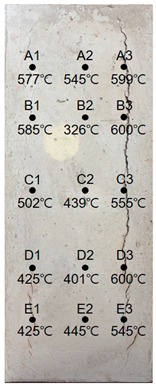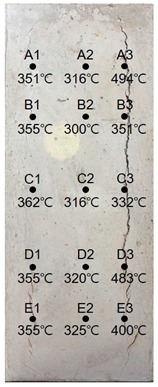# A Method for Instant Estimation of the Temperature Experienced by Fire-Damaged Reinforced Concrete Structures Using Titanium

**DOI:** 10.3390/ma13081993

**Published:** 2020-04-24

**Authors:** Sang-Rak Sim, Dong-Woo Ryu

**Affiliations:** Department of Architecture Engineering, Daejin University, Gyeonggi-do 11159, Korea; simsr@daejin.ac.kr

**Keywords:** concrete, heating temperature, instant estimation of heating temperature

## Abstract

When a concrete structure is exposed to fire, its structural safety is significantly compromised due to the spalling of members and scaling of concrete. In addition, its durability is substantially reduced due to certain chemical changes such as the dehydration of Ca(OH)_2_, the main hydration product of concrete, and the rehydration of CaO. Therefore, when fire damage occurs to a reinforced concrete (RC) building, rapid diagnosis and evaluation techniques are required for immediate repair and reinforcement, requiring a crucial step of quantitatively determining the heating temperature. This study aims to demonstrate a method of estimating the heating temperature experienced by fire damaged RC buildings. The experiments utilized two short RC column specimens with embedded titanium strips. The discoloration characteristics of titanium at high temperatures provided a quick, accurate, and simple mechanism for the estimation of the heating temperature by depth. Empirical equations were derived to estimate the heating temperature as a function of the discoloration characteristics of titanium. Thereafter, a comparison of this estimated temperature with the actual heating temperature measured using thermocouples revealed an average error of less than 20 °C, thereby demonstrating a significantly good correlation and an extremely high reliability of the proposed method.

## 1. Introduction and Background

Concrete, which is the most widely used structural material for buildings, has been generally recognized as typically fire-resistant due to its significantly lower thermal conductivity and thermal diffusion coefficient as compared to those of other structural materials. The degradation of concrete by exposure to high temperatures is, however, known to be severe because of the rapid decrease in its strength when subjected to temperatures higher than 600 °C [1,2]. In particular, when a concrete structure is exposed to fire, direct damage such as exposing of the main reinforcements can occur due to spalling of the members and scaling of concrete, besides its structural safety can get significantly compromised due to some additional damage such as the deformation of beams, buckling of columns, and initiation of shear cracks by thermal expansion [3]. In addition, the cement paste causes a serious impact on the durability of concrete by undergoing chemical changes such as the dehydration of Ca(OH)_2_ at approximately 500 °C and the decomposition of the C–S–H gel at approximately 700 °C. Even after the exposure to fire, the dehydrated CaO reacts with the moisture in the air and is rehydrated to Ca(OH)_2_, whose expanded volume then generates microcracks [4,5,6,7]. Owing to these reasons, when fire damage occurs to an RC structure, timely repair and reinforcement are required. There is, however, no established evaluation criterion that clearly defines the performance degradation extent of concrete structures after a fire. Therefore, a quick and accurate diagnosis and evaluation techniques are required, for which it is important to quantitatively evaluate the heating temperature.

Several studies on the estimation of the heating temperature of fire-damaged concrete have been reported previously. The simplest method is to visually observe the discoloration and properties of the affected surface. When concrete is exposed to high temperatures, it acquires a pink or purple color at 300–600 °C, pale gray at 600–900 °C, and pale yellow at 900–1000 °C due to the oxidation of the iron compounds [8,9,10]. While the above observations can estimate the heating temperature of concrete by quantifying the discoloration characteristics of the concrete surface at high temperatures, this method is limited to the cases, which are composed of 100% ordinary Portland cement (OPC). In recent times when the use of concrete admixtures and composite cements has increased, this method is very impractical because the discoloration characteristics must be calibrated individually for each mix. In addition, the discoloration of the concrete surfaces cannot be observed during a fire due to the black smoke and soot.

As such, in the fire engineering design of Eurocode 2 [11], the 500 °C isotherm method is applied as a simplified assessment method that ignores the strength and elasticity of the part exceeding the 500 °C mark. The Architectural Institute of Japan [12] also presented a method for easily estimating whether the heating temperature of concrete exceeded 500 °C using the neutralization depth measurement method by a phenolphthalein solution. According to a previous study, however, a fire duration of more than three hours is required at 500 °C to completely pyrolyze the Ca(OH)_2_, thus causing the heating temperature to be underestimated when the exposure is less than three hours as the phenolphthalein solution reacts to the remaining traces of Ca(OH)_2_ and exhibits discoloration [13].

Other heating temperature estimation methods include the ultraviolet (UV) spectrum method and the ultrasonic pulse velocity measurement method. Yoshida. M et al. reported a method of estimating the maximum temperature of fire-damaged concrete by measuring changes in the compounds present in the concrete after a fire using UV spectrum [14]. Bo-Tsun Chen et al. reported a method of estimating the heating temperature of concrete through the compressive strength and split tensile strength of concrete as well as using a support vector machine (SVM) on the ultrasonic pulse velocity data [15]. Yang Han et al. reported a method of estimating the maximum temperature experienced by fire-damaged concrete by measuring the acoustic decrepitation of fluid inclusion in the natural silica sand used as the fine aggregate in concrete using the characteristic of fluid inclusion that it is not recoverable when damaged by a fire [16]. The above estimation methods however by a thorough analysis reveal some limitations in being applied to the field because the considerable efforts and time that are required to estimate the heating temperature by depth after collecting core samples from all the fire exposed locations when the fire damaged region is wide. Moreover, when the heating temperature is estimated in a fire-damaged structure using changes in the concrete compounds as indicators, the estimation can be inaccurate because the concrete compounds getting rehydrated due to the water that had been sprinkled for fire suppression. Thus, it is difficult and unreliable to estimate the actual heating temperature using the above methods [17].

Certain studies on the behavior of the steel reinforcements rather than that of the concrete at high temperatures to estimate the heating temperature of fire-damaged RC structures have been reported. Roberto Felicetti et al. compared and evaluated the residual performance of various steel reinforcement types through the M-N envelope [18]. They could approximate the heating temperature at a specific depth of an RC structure where the steel reinforcement was located, but could not determine the heating temperature of the concrete by depth.

Therefore, quantitative estimation of the heating temperature by depth is required to enable timely repair and reinforcement of fire damaged concrete buildings. In particular, considering the pyrolysis temperature of potassium hydroxide, which is 450–550 °C, and the 500 °C isotherm method of Eurocode 2, this study aims to propose a novel method for quick and accurate estimation by setting 400–600 °C as the main goal of heating temperature estimation.

### 1.1. Discoloration Characteristics of Titanium

Titanium metal is silvery white in color under normal conditions. When subjected to high temperatures, it has been reported to exhibit different colors on its surface depending on the temperature. This is because the film thickness on the titanium surface changes and the light reflected from the metal surface causes interference. It has been reported that the color of the metal surface appears yellow at 300 °C, purple at 400 °C, blue at 500 °C, gray at 600–800 °C, and white at 900–1000 °C [19,20,21]. This study aimed to utilize these discoloration characteristics to estimate the heating temperature of concrete by depth.

### 1.2. Color Analysis

There are various methods to express colors, but they can be broadly categorized into two methods; one, the method of expressing in the combinations of red, green, and blue (RGB), and the other in terms of hue, saturation, and brightness (HSB).

The RGB coordinates express the colors by mixing the three primary colors, i.e., red, green, and blue. They express each of these colors using numbers from zero to 255 depending on their respective brightness. This method is referred to as “additive color mixture” because the mixing of the three colors increases the brightness. It, however, has limitations in quantitatively expressing the relationship between the temperature and the discoloration characteristics because the proportions of the mixed colors can be different even for similar colors.

On the other hand, the HSB coordinates are expressed by the hue, saturation, and brightness, where the hue is quantified by angle, the saturation represents the mixing degree of the pure colors with white, and the brightness represents the intensity of light reflected from a material, i.e., relative brightness and darkness [22]. Therefore, the higher the saturation, the closer is the displayed color to the original color; the lower the saturation, the closer the color is to white. Furthermore, the higher the brightness, the closer the color is to the original color; the lower the brightness, the darker it becomes [8].

As such, in this study, the HSB color analysis method was adopted since it is capable of quantitatively expressing the relationship between the temperature and the discoloration characteristics of titanium.

## 2. Materials and Methods

In this study, the discoloration characteristics of titanium metal by temperature were first quantified for the titanium in atmosphere using the HSB color analysis method. Further, regression equations were derived for certain temperature ranges to estimate the heating temperature of the titanium embedded in concrete.

To evaluate the field applicability of the estimated values, short column specimens (dimensions: 600 × 600 × 1500 mm) with embedded titanium metal were fabricated and a fire resistance test was conducted for a duration of three hours as per the ISO 834 Standard time-temperature curve. The heating temperature was then estimated through the color analysis of the recovered titanium metal.

### 2.1. Discoloration Characteristics of Titanium in the Atmosphere

#### 2.1.1. Experiment Overview and Method

In this study, 99.5% pure titanium grade 2 was used. The discoloration characteristics at high temperatures were investigated using an electric furnace.

The target temperature range was set to the 300–700 °C considering that concrete generally exhibits significant strength degradation in terms of structural safety and durability at temperatures higher than 500 °C, as the ratio of residual compressive strength drops below 0.5 and neutralization by heat occurs due to the pyrolysis of Ca(OH)_2_.

In the electric furnace experiment, the discoloration characteristics of titanium metal were investigated when it was heated for one hour at the target temperature. To quantitatively investigate the discoloration characteristics, the top surface of the metal was positioned at a 50-cm height from the floor under a 270-cm high indirect light source condition (fluorescent light with 870 l× illuminance). Images of the metal were captured using a camera fixed at an 80 cm vertical height, and the HSB color coordinate values were measured using the HSB color analysis method.

#### 2.1.2. Experiment Results

**Figure 1:**
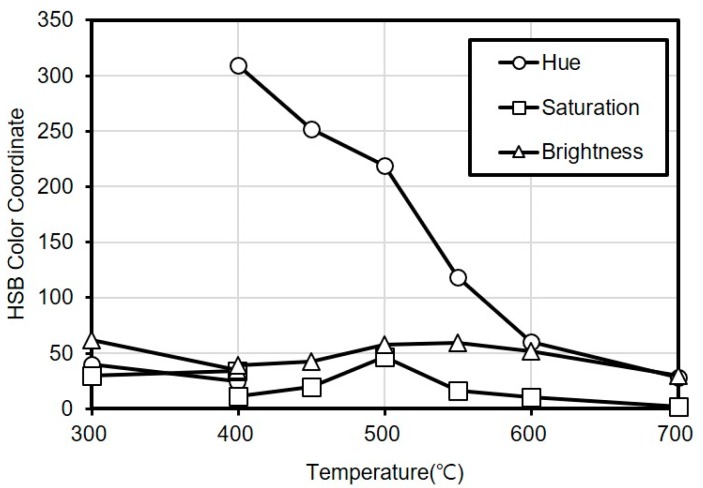
Variations in HSB color coordinates with temperature.

Table 1 shows the results of a visual observation of the discoloration characteristics of titanium through the electric furnace experiment, and Figure 1 shows the results of the quantitative analysis conducted using the color analysis method. The surface of titanium metal turned yellow at 300 °C, purple at 400 and 450 °C, blue at 500 °C, light blue at 550 °C, dark gray at 600 °C and black at 700 °C.

Based on the results of the HSB color analysis, the first-order correlation between the hue (H) that represents the hue angle and the heating temperature could be derived.

In particular, the H value exhibited a clear first-order correlation in the 400–600 °C range, indicating that the heating temperature of concrete by depth can be quantitatively estimated. In addition, at temperatures higher than 600 °C that exhibit gray colors, the discoloration characteristics could be quantified by the saturation (S) that represents color visibility and the brightness (B) that represents light intensity, indicating that the HSB color analysis method is suitable for the heating temperature estimation.

Therefore, in the case of the regression equation derived for estimating the heating temperature of titanium metal, the heating temperature was estimated through the hue angle classified according to the hue in the temperature range of less than 600 °C, and through the brightness, whereby the color of titanium metal turned darker from gray to black, in the temperature range of 600 °C or higher. To estimate the heating temperature, empirical equations, each for a distinct temperature range, were derived as shown in Table 2. The equations were created for the following six ranges: 300–400 °C, 400–450 °C, 450–500 °C, 500–550 °C, 550–600 °C, and 600–700 °C.

### 2.2. Estimation of the Heating Temperature of Concrete

#### 2.2.1. Experimental Plan and Method

After fabricating the short column specimens (600 × 600 × 1500 mm) with embedded titanium metal, air-curing was performed for 91 days. At the age of 91 days, the fire resistance test was performed for three hours based on the ISO 834 Standard Time-Temperature Curve to estimate the heating temperature of concrete by depth.

The heating temperature of titanium metal estimated by the HSB color analysis was compared with the temperature history of the thermocouple embedded at the same position to verify their correlation.

#### 2.2.2. Specimen Specifications

**Figure 2:**
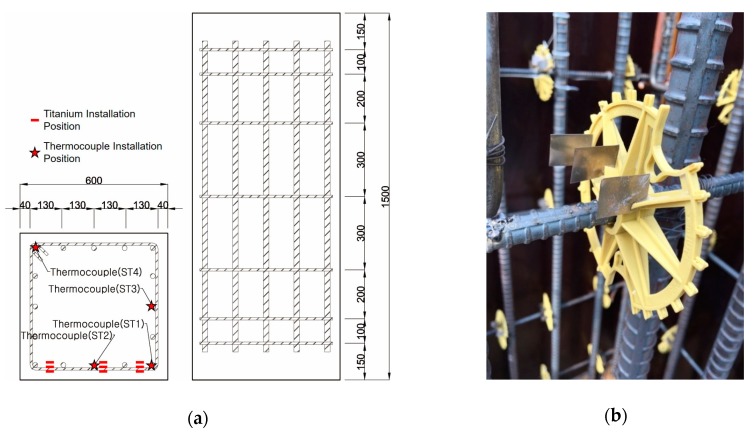
(**a**) Specifications of the RC column specimen; (**b**) titanium metal installation.

The specimens were fabricated having a 600 × 600 × 1500 mm size as represented in Figure 2. D22 rebars were used as the main reinforcements and D10 rebars as the tie bars. The cover thickness was 5 cm.

Four K-type thermocouples were installed at the corners of each specimen and at the center of the main reinforcements. Titanium metal was installed at fifteen positions at 3-, 5-, and 7-cm depths from the front surface of each specimen.

### 2.3. Mix Proportions of Concrete

Table 3 shows the mix proportions of concrete used in this study. OPC (type 1) was used as the cement for the concrete mixes; its properties and composition are listed in Table 4. Blast furnace slag (BS) powder (type 3) and fly ash (FA) (type 2) were used as the admixtures; their properties and composition are presented in Table 5. Furthermore, the strength of the most commonly used normal strength concrete and high strength concrete was set to 24 MPa and 60 MPa, respectively, to investigate the general usability of the temperature estimation of titanium metal. To secure high-strength and fire resistance, polypropylene fibers were added in proportion of 1.0 kg/m^3^ for the 60 MPa specimen. Table 6 shows the Properties of polypropylene fiber used in this study.

### 2.4. Fire Resistance Test

**Figure 3:**
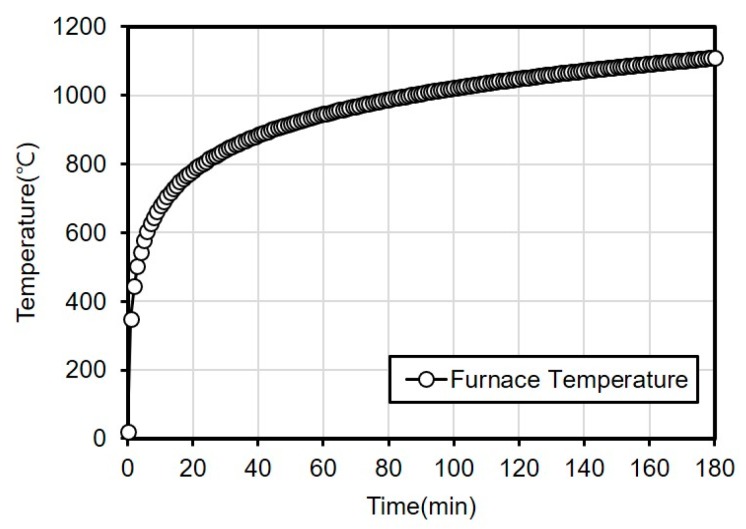
ISO 834 standard time-temperature curve.

**Figure 4:**
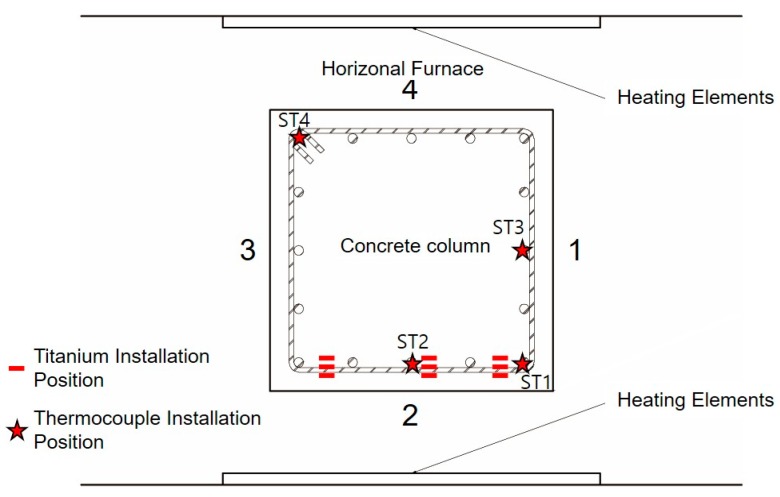
Placement of the short column specimen inside the horizontal furnace.

The fire resistance test was conducted for three hours as per the ISO 834 Standard time-temperature curve shown in Figure 3. Each short column specimen was placed inside the horizontal furnace with the titanium metal facing the heating elements as shown in Figure 4. 

### 2.5. Measurement of the Discoloration Characteristics of Titanium Metal

**Figure 5:**
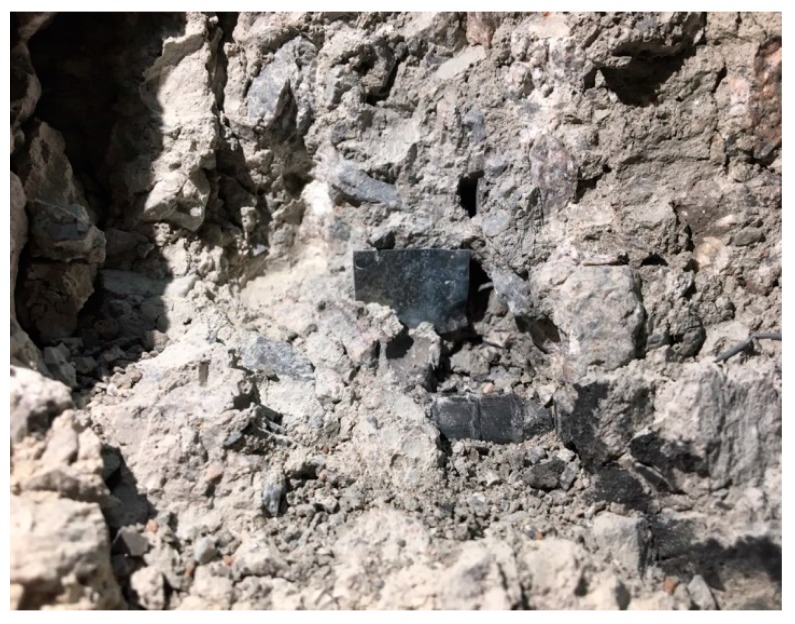
Titanium metal in concrete column.

Upon the completion of the fire resistance test, the embedded titanium metal was recovered; it is shown in Figure 5. To quantitatively investigate the discoloration characteristics of titanium metal, the top surface of the metal was positioned at a 50 cm height from the floor under a 270-cm-high indirect light source condition (fluorescent light with 870 lux illuminance) after the test, and the images of the titanium metal were captured using a camera fixed at a 80 cm vertical height. For the quantitative extraction of the color coordinate values, the Mosaic effect of the popular software Photoshop, which displays the average color of the pixels around a designated spot, was applied to the captured images, and the HSB color coordinate values were measured corresponding to the obtained average value.

Based on the measured color coordinate values, the heating temperatures were estimated using the regression equations derived previously. To examine the accuracy of this estimation, the heating temperatures measured by thermocouples ST1 and ST2, which were installed at a 5-cm depth as shown in Figure 2, were directly compared with those estimated for the titanium probes C2—5 cm and C3—5 cm, respectively.

## 3. Results and Discussion

### 3.1. Results of 24 MPa Specimen

Table 7 shows the estimated heating temperature of the titanium metal embedded in the 24 MPa—class short column specimen through its discoloration characteristics and the color analysis. At a 3-cm depth, the titanium metal turns gray or black at all the positions of rows A to E. At a 5-cm depth, the corners turn blue or green while the centers turn violet or blue. At a 7-cm depth, the center does not show any color change, but the corners turn violet or yellow.

When the color coordinate values of titanium metal were examined, almost all the titanium metal probes at a 3-cm depth showed saturation values less than 8 and brightness values less than 30. Based on these, the heating temperature was estimated using the regression equations. It was implied that the heating temperature reached 700 °C, which is the maximum detectable temperature via discoloration of titanium metal.

At a 5-cm depth, the hue angle values ranged from 35 to 270. Based on this, the heating temperature was estimated to be between 320 and 600 °C.

At a 7-cm depth, the hue angle values ranged from 5 to 260, and thus the heating temperature was estimated to be between 200 and 500 °C.

When the heating temperature of the specimen estimated using titanium discoloration was examined, it was found that the upper sections exhibited higher concrete temperatures for row 1, which was located at the left edge. However, the lower sections exhibited higher temperatures for row 2, which was along the center, and row 3, which was located at the right side edge. The internal temperature distribution within a row was higher for other rows relative to that of row 1. This appears to be because some cover thickness was lost for rows D and E due to the partial loss of material caused by spalling as shown in Table 8.

### 3.2. Results of 60 MPa Specimen

Table 9 shows the estimated heating temperature of titanium metal embedded in the 60 MPa—class short column specimen through its discoloration characteristics and the color analysis. At a 3-cm depth, the titanium metal turns gray or black at all the positions of rows A to E. At a 5-cm depth, the corners turn blue or green while the centers turn yellow or violet. In the case of A1—5 cm, A3—5 cm, and C3—5 cm, one titanium metal strip exhibits various colors, such as violet, blue, and green. At a 7-cm depth, most of the titanium metal strips turn yellow. A3—7 cm and D3—7 cm turn dark blue with violet tint or blue, and E3—7 cm turns violet.

When the color coordinate values of titanium metal were examined, almost all the titanium probes at a 3-cm depth showed saturation and brightness values less than 20. Based on this, the heating temperature was estimated using the regression equations, and it was found that most of the positions exhibited heating temperatures between 650 and 700 °C.

At a 5-cm depth, most of the titanium strips showed hue angle values between 50 and 300. Based on this, the heating temperature was estimated to be between 400 and 550 °C. In addition, for the cases where titanium metal exhibited various colors like at A1—5 cm, C3—5cm, each color was analyzed, and the highest temperature was used as the estimated heating temperature.

At a 7-cm depth, the hue angle values ranged from 30 to 40. Based on this, the heating temperature was estimated to be between 300 and 350 °C. In the case of A3—7 cm, the heating temperature was estimated to be 498 °C because the hue angle value was 220. The heating temperature of D3—7 cm was estimated to be 486 °C because its hue angle value was 228. In the case of E3—7 cm, the hue angle value was 300, and thus the heating temperature was estimated to be 407 °C.

It was observed that the internal temperature distribution within row 3 was higher than that of row 1. This appears to be because of a crack with a 24.68 mm width occurring on the surface of the specimen where row 3 is located (see Table 10) and the flames had possibly infiltrated through the crack. 

### 3.3. Comparison between the Estimated and the Measured Temperatures

**Figure 6:**
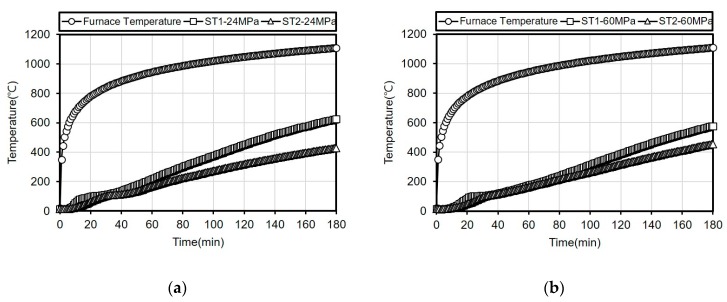
Main reinforcement temperature history (**a**) 24 MPa; (**b**) 60 MPa.

The temperature history of the main reinforcements (at a 5-cm depth from the surface) of each specimen was obtained from the three-hour fire resistance test conducted based on the ISO 834 Standard time-temperature curve. Figure 6 shows the main reinforcement temperature histories obtained from the thermocouples.

When the temperature history of the 24 MPa specimen is examined, the main reinforcement temperature at position ST1 is observed to be 624 °C and the estimated heating temperature of titanium metal C3—5 cm at the same position was 600 °C, exhibiting a difference of approximately 24 °C. Further, the main reinforcement temperature at position ST2 is observed to be 428 °C while the estimated heating temperature of titanium metal C2—5 cm at the same position was 420 °C, exhibiting a difference of approximately 8 °C.

When the temperature history of the 60 MPa specimen is examined, the main reinforcement temperature at position ST1 is observed to be 577 °C while the estimated heating temperature of titanium metal C3—5 cm at the same position was 550 °C, exhibiting a difference of approximately 27 °C. The main reinforcement temperature at position ST2 is observed to be 454 °C and the estimated heating temperature of titanium metal C2—5 cm at the same position was 439 °C, revealing a difference of approximately 15 °C.

For both specimens, the temperature differences at position ST1 are larger than that at position ST2. This appears to be because of the position of the main reinforcement thermocouple (ST1) being relatively closer to the edge than the titanium metal C3—5 cm and thus the temperature increment is larger.

Furthermore, when the main reinforcement temperatures of concrete are compared, the result confirms that the difference between the two specimens of 24 MPa and 60 MPa is not significant.

As the strength of concrete increases, the thermal conductivity increases because the porous structure becomes tighter. However, in the case of fiber-mixed concrete, as in the study result of Won, JP. et al. [23], the temperature difference is not significant because the thermal conductivity decreases due to the melting of fiber at high temperatures.

## 4. Conclusions

The color change of the embedded titanium metal in the three-hour fire resistance test conducted with short column specimens was analyzed through HSB color coordinate values, and the results were as follows.

(1) When the internal heating temperature of high-strength concrete was estimated by depth, the temperature ranged from 600 to 700 °C at a 3-cm depth from the surface, from 320 to 550 °C at a 5-cm depth, and from 250 to 480 °C at a 7-cm depth. A comparison with the actual heating temperature measured using thermocouples revealed a difference of 15 °C, thereby demonstrating a considerably good correlation.
(2) It was observed that the corners and upper sections of the specimens exhibited higher temperatures. This appears to be because of the proximity of the corners to the surfaces on both sides that contain them while the upper sections were affected by convection caused by heating.
(3) It was successfully demonstrated that by using the discoloration characteristics of titanium, it is possible to estimate the heating temperature by depth quantitatively, quickly, and accurately. The reliability was confirmed by the established correlation between the estimated and the measured temperatures for both the specimens.

## Figures and Tables

**Table 1:** Variations in color of titanium metal with increasing temperature.

Temperature	300 °C	400 °C	450 °C	500 °C	550 °C	600 °C	700 °C
Color change of titanium metal	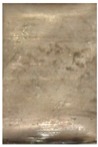	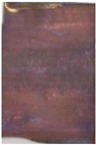	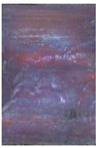	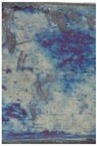	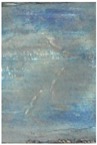	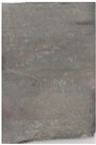	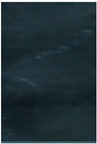

**Table 2:** Change in color of titanium metal with temperature.

Temperature Range	300–400 °C	400–450 °C	450–500 °C	500–550 °C	550–600 °C	**600–700** °C
Equation	$T=\frac{1700-20H}{3}$	$T=\frac{38250-50H}{57}$	$T=\frac{27450-50H}{33}$	$T=\frac{61450-50H}{101}$	$T=\frac{18975-25H}{29}$	$T=\frac{6400-50S}{9}$
Condition	H<60	252<H≤60	219<H≤252	130<H≤219	60<H≤130	S<20 and B<30

**Table 3:** Mix proportion of concrete.

Specimens (MPa)	W/B (%)	S/a (%)	W (kg/m^3^)	Weight (kg/m^3^)						
				OPC	BS	FA	G	S1	S2	PP
24	55.3	53.9	177	180	96	44	845	395	592	
60	32.1	44.8	180	365	112	84	894	290	434	1.0

W/B: Water Binder ratio, S/a: Sand to Aggregate ratio, OPC: Ordinary Portland Cement. GGBS: Ground Granulated Blast Furnace Slag, FA: Fly Ash, G: Gravel, S1: Sea sand, S2: Crushed sand.

**Table 4:** Physical and chemical properties of OPC.

Physical Properties			Chemical Proportion (%)					
Density (g/cm^3^)	Blaine (cm^2^/g)	Ignition Loss (%)	SiO_2_	Al_2_O_3_	Fe_2_O_3_	CaO	MgO	SO_3_
3.14	3149	0.79	22.0	5.27	3.44	63.4	2.13	1.96

**Table 5:** Physical and chemical properties of mineral admixture.

Type	Density (g/cm^3^)	Activity Factor (%)			Component		Ignition Loss (%)	Blaine (cm^2^/g)
		Age 7 d	Age 28 d	Age 91 d				
FA	2.20	-	90	96	CaO	SiO_2_	2.8	3707
BS	2.89	86.9	110	111	MgO	SO_3_	0.75	4231

**Table 6:** Properties of polypropylene fiber.

Length	Diameter	Specific Gravity	Melting Point	Tensile Strength
6.0 mm	20 µm	0.91	160–170 °C	500 MPa or more

**Table 7:** Discoloration results of titanium metal by depth—24 MPa specimen.

Thickness of Cover Concrete		3 cm			5 cm			7 cm		
		1	2	3	1	2	3	1	2	3
A	Image	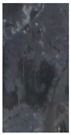	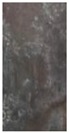	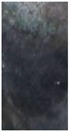	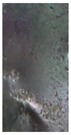	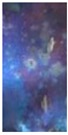	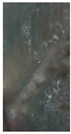	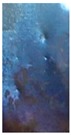	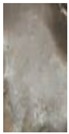	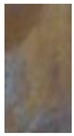
	HSB	25, 7, 30	28, 5, 32	27, 7, 32	200, 9, 29	237, 34, 45	93, 9, 25	241, 42, 67	45, 6, 67	38, 31, 49
	Heat Temperature	700 °C	700 °C	700 °C	600 °C	469 °C	600 °C	466 °C	200 °C	305 °C
B	Image	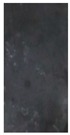	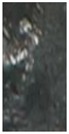	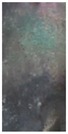	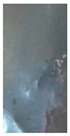	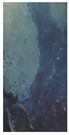	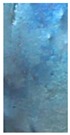	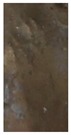	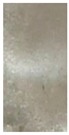	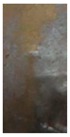
	HSB	243, 7, 27	110, 3, 27	235, 4, 35	183, 20, 30	223, 28, 36	205, 28, 57	37, 29, 32	45, 14, 56	33, 21, 22
	Heat Temperature	700 °C	700 °C	700 °C	530 °C	480 °C	500 °C	310 °C	200 °C	320 °C
C	Image	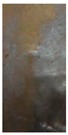	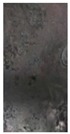	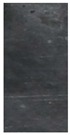	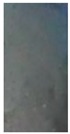	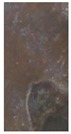	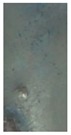	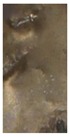	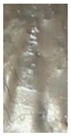	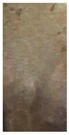
	HSB	236, 7, 24	30, 4, 32	228, 6, 29	186, 25, 40	290, 24, 30	123, 10, 31	39, 29, 43	50, 12, 60	39, 25, 53
	Heat Temperature	700 °C	700 °C	700 °C	530 °C	420 °C	546 °C	302 °C	200 °C	302 °C
D	Image	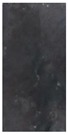	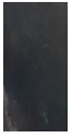	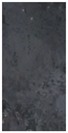	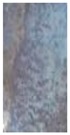	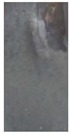	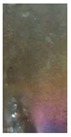	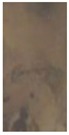	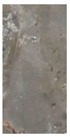	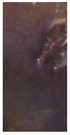
	HSB	232, 5, 23	234, 3, 24	229, 7, 32	223, 16, 45	208, 22, 30	69, 10, 42	35, 25, 29	33, 29, 43	5, 25, 27
	Heat Temperature	700 °C	700 °C	700 °C	497 °C	495 °C	550 °C	316 °C	320 °C	399 °C
F	Image	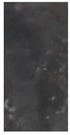	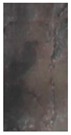	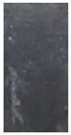	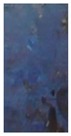	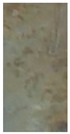	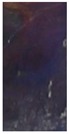	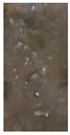	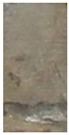	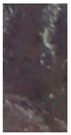
	HSB	14, 2, 36	17, 8, 34	232, 7, 32	224, 34, 47	120, 25, 50	250, 47, 51	34, 20, 37	42, 20, 52	312, 23, 28
	Heat Temperature	700 °C	700 °C	700 °C	481 °C	557 °C	453 °C	317 °C	200 °C	400 °C

**Table 8:** Results of estimated temperature distribution inside the specimen by depth—24 MPa specimen.

24 MPa		
3 cm	5 cm	7 cm
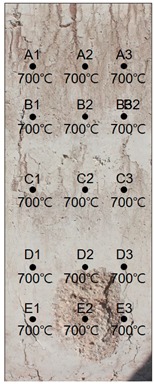	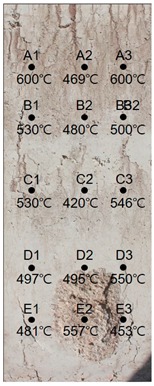	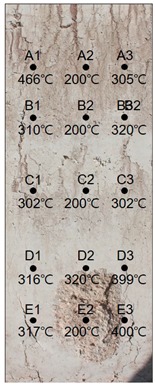

**Table 9:** Discoloration results of titanium metal by depth—60 MPa specimen.

Thickness of Cover Concrete		3 cm			5 cm			7 cm		
		1	2	3	1	2	3	1	2	3
A	Image	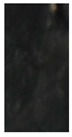	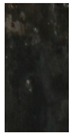	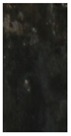	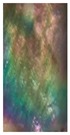	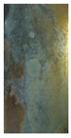	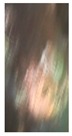	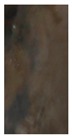	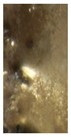	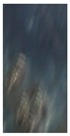
	HSB	112, 3, 14	68, 2, 16	67, 2, 19	98, 28, 53	122, 29, 47	73, 17, 46	33, 24, 29	44, 38, 55	219, 23, 29
	Heat Temperature	700 °C	700 °C	700 °C	577 °C	545 °C	599 °C	351 °C	316 °C	494 °C
B	Image	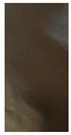	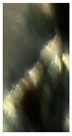	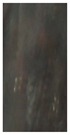	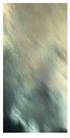	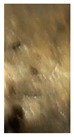	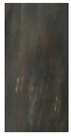	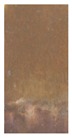	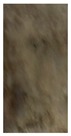	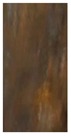
	HSB	36, 4, 19	68, 2, 16	45, 2, 25	89, 12, 58	41, 41, 45	179, 9, 36	33, 24, 29	43, 26, 46	33, 56, 40
	Heat Temperature	675 °C	700 °C	700 °C	585 °C	326 °C	600 °C	355 °C	300 °C	351 °C
C	Image	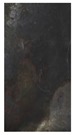	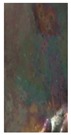	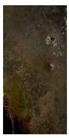	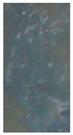	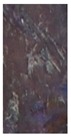	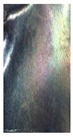	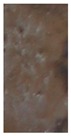	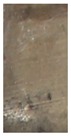	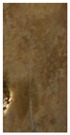
	HSB	26, 3, 33	25, 20, 34	38, 18, 44	202, 15, 40	266, 17, 33	111, 12, 88	27, 21, 40	38, 23, 52	39, 45, 45
	Heat Temperature	687 °C	649 °C	700 °C	502 °C	439 °C	555 °C	362 °C	316 °C	332 °C
D	Image	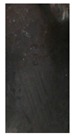	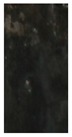	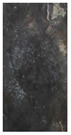	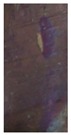	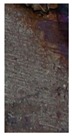	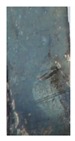	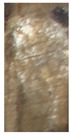	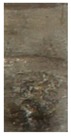	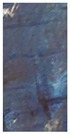
	HSB	112, 2, 14	68, 7, 16	67, 8, 19	278, 23, 32	300, 19, 23	179, 9, 39	33, 24, 29	43, 26, 46	228, 25, 43
	Heat Temperature	675 °C	637 °C	700 °C	425 °C	401 °C	511 °C	355 °C	320 °C	483 °C
F	Image	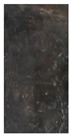	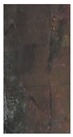	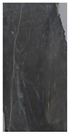	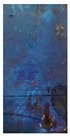	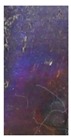	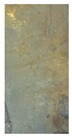	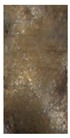	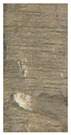	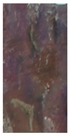
	HSB	34, 5, 25	39, 9, 52	40, 2, 15	278, 23, 32	253, 47, 51	61, 17, 59	33, 24, 29	42, 22, 56	300, 16, 34
	Heat Temperature	662 °C	612 °C	700 °C	425 °C	445 °C	545 °C	355 °C	325 °C	400 °C

**Table 10:** Results of estimated temperature distribution inside the specimen by depth—60 MPa specimen.

24 MPa		
3 cm	5 cm	7 cm